# The Effects of Employment on Depression and Life Satisfaction Among Old-Aged Using the DD Method Combined With PSM

**DOI:** 10.1093/geroni/igaa057.1315

**Published:** 2020-12-16

**Authors:** Si Young Song, Hey Jung Jun, Sun Ah Lee

**Affiliations:** Yonsei University, Seoul, Republic of Korea

## Abstract

The purpose of this study is to explore the effect of employment on depression and life satisfaction among old-aged. Using 12th (2017) wave and 13th (2018) wave of Korean Welfare Panel Study (KoWePS), three stages of analyses were conducted. First, through propensity score matching (PSM) method, sample with similar propensity scores was matched between the group that did not work in 12th wave but worked in 13th wave (experimental group, N=180), and the group that did not work in 12th and 13th wave (comparative group, N=180). Second, the matched sample was used to conduct multiple regression analysis with the group dummy variable (experimental group, comparative group) as an independent variable, and depression and life satisfaction as the dependent variables. Third, combined model of propensity score matching (PSM) and double difference (DD) method was conducted to more appropriately derive the net effect of employment. The results of multiple regression after propensity matching showed that employment had a positive effect on reducing depression (B= -1.70, p< .01) and increasing life satisfaction (B= .12, p< .01) in old-aged. Furthermore, in combined model of PSM and DD, life satisfaction was improved when employed compared to non-employed (B= .15, p< .05). The results of this study are meaningful in that the meaning of employment in old-aged is more clearly derived by solving selection bias and endogenous problems. Also, this study may provide reference for establishing welfare policies related to employment among old-aged.

